# Supplementary material for: Thiol-ene Reaction: An Efficient Tool to Design Lipophilic Polyphosphoesters for Drug Delivery Systems
Source: Molecules. 2021 Mar 20;26(6):1750. doi: 10.3390/molecules26061750 (PMC8003835; doi:10.3390/molecules26061750)
Supplement: Supplementary file 1 [file molecules-26-01750-s001.pdf]

Supporting information

Thiol-ene reaction: an efficient tool to design lipophilic polyphosphoesters for drug delivery systems

Stéphanie Vanslambrouck, Raphaël Riva, Bernard Ucakar, Véronique Préat, Mick Gagliardi, Daniel G. M. Molin, Philippe Lecomte and Christine Jérôme

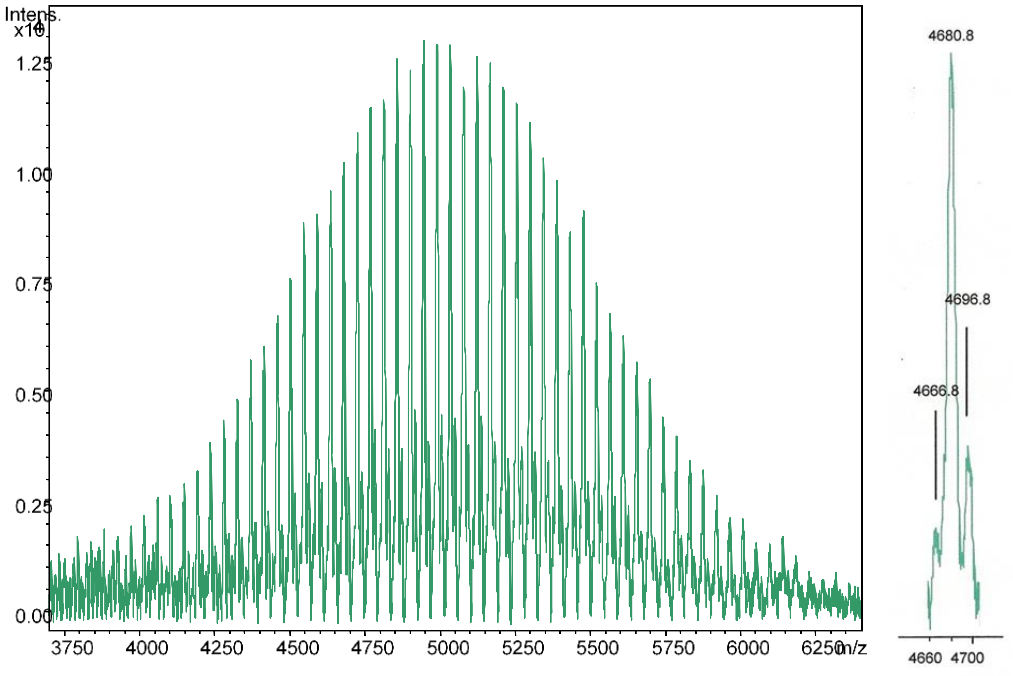

Figure S1. MALDI-TOF spectrum of the starting MeO-PEG-OH macroinitiator. On the right, the zoom of the 4660-4700 range shows peaks at 4680.8 and 4696.8 corresponding to the expected CH_3-O-PEG-OH cationized with sodium and potassium, respectively. The peak at 4666.8 (i.e. 14 mass units below the main peak) corresponds to the HO-PEG-OH present in the commercial product.

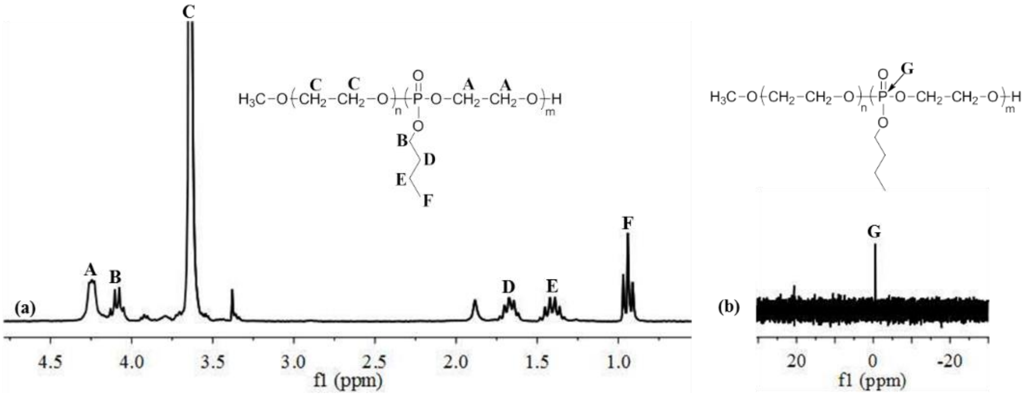

Figure S2. (a)^{1}H NMR and (b) ^{1}H-decoupled ^{31}P NMR spectra of PEG-b-PButEP

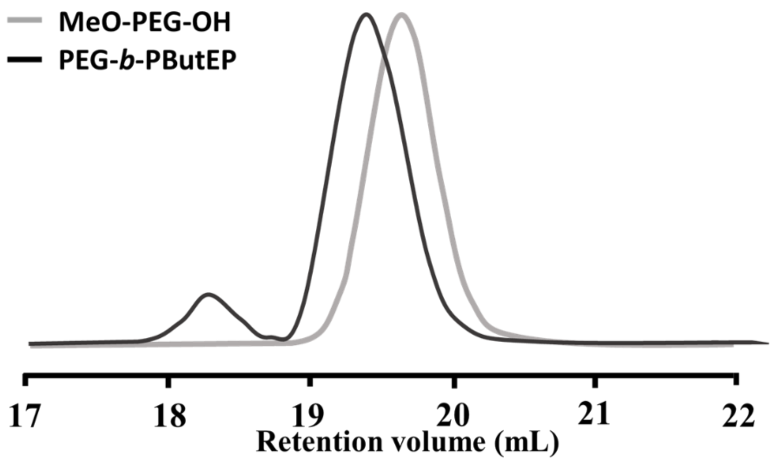

Figure S3. SEC traces in THF of PEG-b-PButEP (black curve) and the starting MeO-PEG-OH (grey curve).

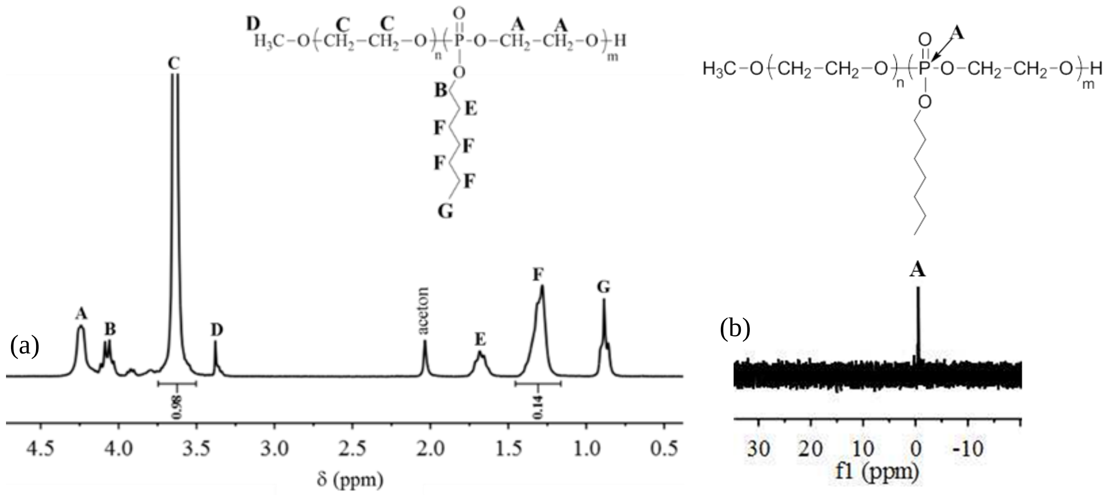

Figure S4. (a)^{1}H NMR and (b) ^{1}H-decoupled ^{31}P NMR spectra of PEG-b-PHEP

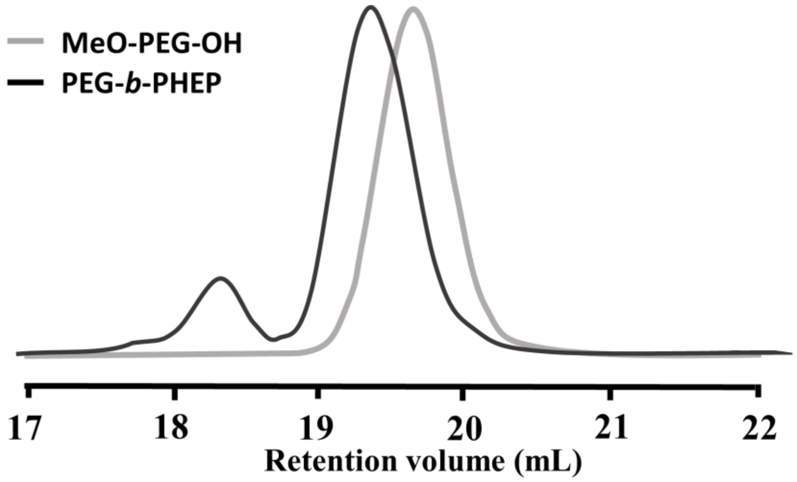

Figure S5. SEC traces in THF of PEG-b-PHEP (black curve) and the starting MeO-PEG-OH (grey curve).
